# Graves' Ophthalmopathy: VISA versus EUGOGO Classification, Assessment, and Management

**DOI:** 10.1155/2015/249125

**Published:** 2015-08-17

**Authors:** Jesús Barrio-Barrio, Alfonso L. Sabater, Elvira Bonet-Farriol, Álvaro Velázquez-Villoria, Juan C. Galofré

**Affiliations:** ^1^Department of Ophthalmology, Clínica Universidad de Navarra, Navarra Institute for Health Research (IdiSNA), 31008 Pamplona, Spain; ^2^Department of Endocrinology and Nutrition, Clínica Universidad de Navarra, Navarra Institute for Health Research (IdiSNA), 31008 Pamplona, Spain

## Abstract

Graves' ophthalmopathy (GO) is an autoimmune inflammatory disorder associated with thyroid disease which affects ocular and orbital tissues. GO follows a biphasic course in which an initial active phase of progression is followed by a subsequent partial regression and a static inactive phase. Although the majority of GO patients have a mild, self-limiting, and nonprogressive ocular involvement, about 3–7% of GO patients exhibit a severe sight-threatening form of the disease due to corneal exposure or compressive optic neuropathy. An appropriate assessment of both severity and activity of the disease warrants an adequate treatment. The VISA (vision, inflammation, strabismus, and appearance), and the European Group of Graves' Orbitopathy (EUGOGO) classifications are the two widely used grading systems conceived to assess the activity and severity of GO and guide the therapeutic decision making. A critical analysis of classification, assessment, and management systems is reported. A simplified “GO activity assessment checklist” for routine clinical practice is proposed. Current treatments are reviewed and management guidelines according to the severity and activity of the disease are provided. New treatment modalities such as specific monoclonal antibodies, TSH-R antagonists, and other immunomodulatory agents show a promising outcome for GO patients.

## 1. Introduction

Graves' ophthalmopathy (GO), thyroid eye disease (TED), or thyroid associated orbitopathy (TAO) is an immunomediated inflammatory disorder that produces expansion of the extraocular muscles and fat in the orbit. Edema, accumulation of glycosaminoglycans and collagen, and adipogenesis cause most patients to have enlargement of both extraocular muscle and orbital adipose tissue with a predominance of either one in some of the patients [1, 2]. GO is the most common and most important extrathyroidal manifestation of Graves' disease (GD) [3]. This condition generally occurs in patients with Graves' hyperthyroidism but sometimes may take place in patients with euthyroid or hypothyroid autoimmune thyroiditis [1]. The estimated incidence of GO is 16 women or 3 men per 100,000 person per year [4].

GD is an autoimmune disorder where loss of immunological tolerance to the thyroid-stimulating hormone receptor (TSH-R) is pivotal to the appearance of the specific antibodies [5, 6]. Several findings in patients with GO, including elevated TSH-R expression in orbital tissues and elevated levels of TSH-R antibodies, support the concept that the TSH-R is the primary autoantigen in GO [1, 7]. This concept has recently been reinforced with the development of the first complete animal model of GO achieved by immunization of female BALB/c mice with human TSHR A subunit [8]. The serum levels of TSH-R autoantibodies correlate positively with the clinical features of GO. These constitute an independent risk factor and help predict the severity and progression of the disease [1].

The aim of this review is to present a comprehensive and concise overview of the current concepts needed to evaluate GO patients and to provide guidelines to manage this disease.

## 2. Pathophysiology of Graves' Ophthalmopathy

The pathogenesis of this illness is summarized in three main phenomena: (1) inflammation of the periorbital soft tissues; (2) overproduction of glycosaminoglycans by orbital fibroblasts; and (3) hyperplasia of adipose tissue [1]. Along with the orbital fibroblasts, the perimysium fibroblasts proliferate, producing collagen and glycosaminoglycans in the extracellular matrix. The polyanionic charge and the extremely high osmotic pressure of this matrix substance render it highly hydrophilic and increase its capacity to retain water. As a consequence, the extraocular muscles swell dramatically [9]. Several clinical manifestations of GO are caused by an increase in the orbital soft tissue volume which leads to a higher pressure within the inexpandable bone cavity. The periorbital edema is primarily congestive and it probably reflects a decrease in venous draining due to compression in the orbital space [10]. Conversely, development of new fat cells (adipogenesis) is also a cause of increased orbital tissue volume. The orbit contains different subpopulations of fibroblasts exhibiting phenotypic heterogeneity. This implies important functional consequences from the cellular diversity and provides evidence suggesting divergent biological roles for fibroblasts within the extraocular muscles and fibroblasts from the adipose tissue. The first ones, when exposed to cytokines, can differentiate into myofibroblasts and then participate in inflammation, repair, and fibrosis. On the other hand, half of the fibroblasts within the adipose tissue are preadipocytes that, under certain conditions, can be induced to differentiate. When prompted by the constellation of growth factors and cytokines that are expressed as a consequence of GO, these cells may undergo differentiation into adipocytes and thus contribute to the increased tissue volume associated with the disease [11, 12].

In most cases, GO develops with only one inflammatory onset (active phase), which is followed by a phase of stillness (inactive phase). In the inactive phase, the long lasting muscular edema along with the increased production of collagen leads ultimately to atrophy, fibrosis, and sclerosis of the extraocular musculature and subsequently to restrictive strabismus.

## 3. Clinical Features of Graves' Ophthalmopathy

The GO diagnosis is typically made clinically based on presenting ocular symptoms and signs. Timely diagnosis permits appropriate evaluation and treatment and might prevent the progression to more severe disease manifestations.

Nearly 50% of patients with GD report symptoms of ophthalmopathy, which are generally mild [13]. Symptoms are related to (1) ocular exposure (ocular dryness and grittiness, photophobia, excessive tearing, and blurred vision), (2) periorbital soft tissue inflammation and congestion (sensation of retroocular pressure, conjunctival redness, and eyelid swelling), or (3) extraocular muscle involvement (aching with eye movement, restricted ocular motility, and double vision).

The two most common signs of GO are upper eyelid retraction (90% of patients) and proptosis [1, 14]. Upper eyelid retraction is produced by levator/Müller muscle inflammation and fibrosis or by levator complex overaction secondary to inferior rectus restriction (pseudo-lid retraction). Proptosis is caused by expansion of the orbital fat and/or muscles. Also, the lacrimal gland is frequently involved and enlarged [15].

About 3–7% of GO patients exhibit a severe sight-threatening form of the disease due to corneal exposure or compressive optic neuropathy [1, 2]. Dysthyroid optic neuropathy (DON) is commonly caused by enlarged extraocular muscles at the orbital apex compressing the optic nerve. Symptoms typically consist of desaturation of colors and blurring of central vision. Afferent pupil defect and optic disc edema are specific signs but are not always present. This optic neuropathy is potentially reversible with appropriate treatment. It is more frequently developed by males, elderly, and diabetic patients [2].

If the clinical features are sufficient, orbital imaging may not be necessary for diagnosis of GO. In any case, computed tomographic scans show that most patients with GO have enlargement of both the orbital fat compartments and the extraocular muscles and that others appear to have involvement of only the adipose tissue or extraocular muscle. Type 1 orbitopathy (lipogenic variant) or type 2 orbitopathy (myogenic variant) is differentiated depending on which component is predominant [16]. Calculation of orbital soft tissue volumes may be helpful in understanding the etiology and pathogenesis of the disease and permits an assessment of natural progression or response to therapy [17]. CT scans are also useful to demonstrate the enlarged extraocular muscles crowding the optic nerve at the orbital apex when optic neuropathy is suspected and to plan an orbital bone decompression surgery when necessary. Comerci et al. [18] have developed an MRI-based computer-assisted segmentation method so that the volumes of fat, muscle, and vitreous bodies are automatically calculated for each orbital quadrant of each eye accordingly. Regional automatic assessment of intraorbital fat by dividing the orbits into four quadrants could be useful for more accurate surgical planning and for follow-up studies. Magnetic resonance imaging with diffusion-weighted imaging (DWI) has recently been proven to be useful in the objective assessment of activity in GO patients [19].

As mentioned, typically GO follows a biphasic course. Active phase generally lasts for 18–36 months, followed by a stable or inactive phase. It is therefore essential to differentiate between the concepts of* activity* (refers to the inflammatory process) and* severity* (refers to the quality of life or the risk of vision loss) [20]. The currently assessment protocols of GO have, on one hand, specific scores to evaluate the activity and, on the other hand, items to evaluate the severity of the disease.

## 4. Assessment Protocols of Graves' Ophthalmopathy

Several classification systems have been conceived to assess the clinical manifestations of GO. In 1969, Werner reported the NO SPECS Classification (No physical signs or symptoms, Only signs, Soft tissue involvement, Proptosis, Extraocular muscle signs, Corneal involvement, and Sight loss) [21]. The modified NO SPECS was also published by Werner in 1977 and has been broadly used since then [22]. This classification grades exclusively for clinical* severity* and does not provide a means of distinguishing inflammatory progressive from noninflammatory stationary Graves' ophthalmopathy (Table 1). Therefore, the indication for treatments used to be based exclusively in the severity of symptoms instead of the rate of progression of the disease until 1989, when Mourits et al. described the Clinical Activity Score (CAS) [23]. This score based on the classical signs of acute inflammation (pain, redness, swelling, and impaired function) was proposed as a clinical classification to discriminate easily between the active and quiescent stages of the disease and was modified in 1997 [24] (Table 2).

The currently grading systems used for the assessment of GO are the VISA Classification (vision, inflammation, strabismus, and appearance) [2, 25] and the European Group of Graves' Orbitopathy (EUGOGO) Classification [3]. Both systems are grounded in the NO SPECS and CAS classifications and use indicators to assess the signs of* activity* and the degree of* severity*. More importantly, they allow the clinician to guide the treatment of the patient with GO. VISA is more commonly used in North America and Canada while EUGOGO is in Europe. Since the two protocols are not interchangeable, only one of them should be employed as a reference in a specific patient.

### 4.1. VISA Classification

The VISA system was developed by Dolman and Rootman in 2006 [25] and adopted with modifications by the International Thyroid Eye Disease Society (ITEDS). The current version is designed for office use and can be downloaded from the ITEDS website (http://www.thyroideyedisease.org/). The VISA system is based on symptoms and signs inputs. The system assesses 4 severity parameters: V (vision); I (inflammation/congestion); S (strabismus/motility restriction); and A (appearance/exposure). Each feature is considered and graded independently. A global severity grade (maximum score is 20 points) is the sum of each of the involved systems graded independently: vision: 1 point; inflammation/congestion: 10 points; strabismus: 6 points (diplopia: 3 points plus restriction: 3 points); appearance/exposure: 3 points.

**Figure 1:**
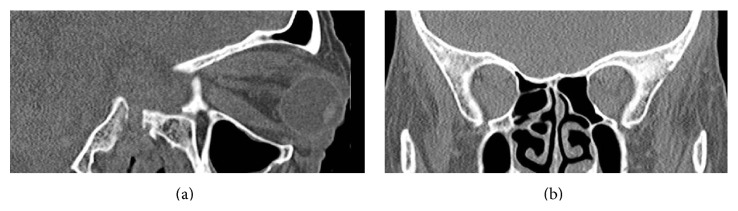
(a) Sagittal CT scan showing severe exophthalmos. (b) Coronal CT scan: apical crowding causing bilateral DON.

*Vision (V)* evaluates the visual repercussion particularly due to the development of dysthyroid optic neuropathy. This is assessed through visual acuity, pupillary reflexes, color vision, visual fields, optic nerve examination, and visual evoked potentials. Most of these tests should be performed in all patients, as optic neuropathy frequently occurs in patients with little or no proptosis. CT scans may be necessary in selected cases to confirm the presence of an orbital apex syndrome or before surgical decompression (Figure 1).

**Figure 2:**
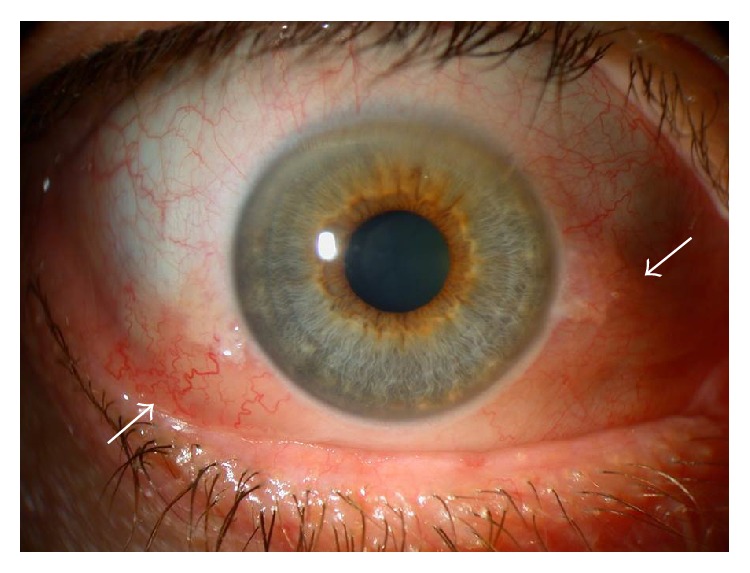
Chemosis. Notice the conjunctiva separated from the sclera and behind the grey line (arrows) and diffuse conjunctival redness.

Soft tissue* inflammation/congestion (I)* evaluation is graded according to the worst score for the eye or the eyelid with the Inflammatory Index (Table 3). Symptoms include orbital ache at rest or with ocular movement and diurnal variation (inflammation worsening with the head dependent after sleep or worsening of diplopia at morning). Signs include caruncular edema, chemosis, conjuntival redness, lid redness, and lid edema. Chemosis is graded as 1 if the conjunctiva lies behind the grey line of the lid (Figure 2) and as 2 if it extends anterior to the grey line. Lid edema is graded as 1 if it is present but not causing overhanging of the tissues and as 2 if it causes a roll in the lid skin including festoons in the lower lid. Cases with moderate inflammatory index (less than 4 of 10) are managed conservatively. Patients with high scores (above 5 of 10) or with subjective or objective evidence of progression in the inflammation are offered a more aggressive therapy.

The presence of* strabismus/motility restriction (S)* is documented by three aspects. (1) Diplopia that is graded from 0 to 3 (0 = no diplopia, 1 = diplopia with horizontal or vertical gaze, 2 = intermittent diplopia in straight gaze, and 3 = constant diplopia in straight gaze). Fluctuation of diplopia with worsening in the morning is frequent during the active phase of the disease. (2) Ocular ductions are measured to the nearest 5° in four directions using the corneal light reflex technique. Accurate assessment of changes in ocular ductions in GO is vital to identify progressive disease, management, and response to therapy assessment. Any change of ≥12° in any direction can be considered progression [26]. (3) Ocular restriction can be graded from 0 to 3 based on the range of ductions (0 = duction >45°, 1 = 30–45°, 2 = 15–30°, and 3 < 15°). Strabismus can be quantified by prism cover testing in order to plan surgical treatment.

In the assessment of the* appearance/exposure (A)* symptoms include appearance concerns (such as bulging eyes, eyelid retraction, and fat pockets) and those derived from ocular exposure (such as gritting sensation, photophobia, dryness, and secondary tearing). Signs include measurements of eyelid retraction (millimeters from the pupillary light reflex to the lid margin); scleral show (millimeters from the limbus to the lid margin); levator palpebrae superioris function; lagophthalmos (incomplete eyelid closure); and proptosis with the Hertel exophthalmometer. Signs of corneal exposure are best assessed with the slit-lamp microscope and may include punctate epithelial erosions, ulcerations, and, in severe cases, corneal thinning and risk of perforation.

### 4.2. EUGOGO Classification

The EUGOGO was established in 1999 [20]. The Europeans developed an assessment protocol for the evaluation of patients with GO based upon activity and severity parameters. The disease activity is evaluated based on the modified Clinical Activity Score (CAS) [24]. Some severity parameters are evaluated by comparison with an image atlas developed by the group itself. New patient and follow-up forms, together with the color atlas, may be downloaded from the EUGOGO website (http://www.eugogo.eu/). A practical classification for the management of the ophthalmopathy according to its severity was also developed [3].

#### 4.2.1. EUGOGO Activity Measures of Graves' Ophthalmopathy: Clinical Activity Score

Disease activity is assessed through the rating of the 10 items of the modified CAS (Table 2). This Clinical Activity Score is based on four of the five well-known classical signs of inflammation (pain, redness, (warmth), swelling, and impaired function). For each of the 10 items present, one point is given. Each item has the same weight. The sum of these points is the CAS which ranges from 0 to 10.

**Figure 3:**
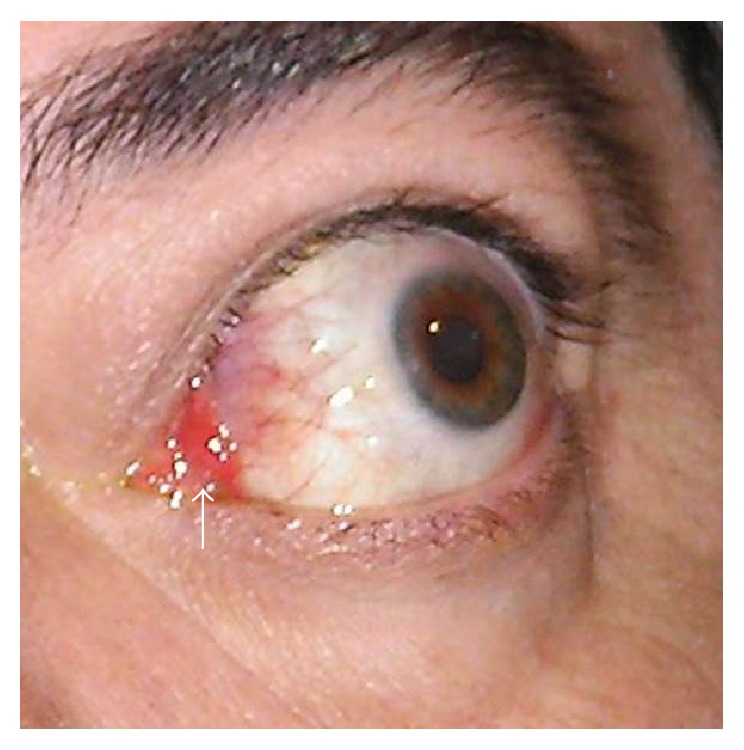
Inflammation of the plica (arrow) with diffuse conjunctival redness.

Soft tissue inflammatory signs and symptoms (pain, redness, and swelling) are graded with the first 8 items. Orbital pain (spontaneous or gaze evoked) should only be scored if present for more than a few seconds and more often than just occasionally. EUGOGO atlas is of great help in evaluating soft tissue inflammatory signs. Only eyelid swelling and eyelid erythema thought to be due to active GO should be scored. When swelling or erythema varies between upper and lower eyelid of an eye the more severe lid should be used to score that eye. Only “moderate” or “severe” and not “mild” eyelid swelling should be recorded as CAS positive. Some of the signs, such as redness of the conjunctiva, may be difficult to recognize because of its nonspecificity. It should be assessed without slit-lamp at 1 meter from the patient. Only redness due to active GO should be scored: diffuse redness, covering at least one quadrant. Redness of the conjunctiva as a result of corneal stippling or ulceration is not what is considered a sign of active inflammation of the orbital tissues [24]. “Equivocal” or “mild” conjunctival redness should not be given a CAS score. Chemosis is assessed with slit-lamp at 60° midway between the limbus and the lateral canthus; true chemosis (separation of conjunctiva from sclera present in >1/3 of the total height of the palpebral aperture or conjunctiva prolapsing anterior to grey line of eyelid) should be distinguished from the redundant folds of the conjunctiva (conjunctivochalasis, CAS negative). If plica is prolapsed through closed eyelids or caruncle and/or plica are inflamed (Figure 3), CAS should be recorded as positive. Increasing proptosis ≥ 2 mm in the previous 1 to 3 months is the ultimate item to evaluate swelling [27].

Impaired function is graded with the last 2 items: decreasing uniocular excursion in any one direction of ≥ 8° and decrease in visual acuity equivalent to 1 Snellen line in the previous 1 to 3 months. During the first visit, the first 7 items are assessed, resulting in an active ophthalmopathy if the total score is higher than or equal to 3/7. In the follow-up visits, the CAS is assessed on the 10 items, and the ophthalmopathy is considered to be active if the score is higher than or equal to 4/10.

#### 4.2.2. EUGOGO Severity Measures of Graves' Ophthalmopathy

Severity assessment (Table 4) is based on the following:

1. Evaluation of CAS items for soft tissue inflammation except pain that is not taken into account is done. Eyelid swelling is classified as mild, moderate (definite subcutaneous fluid or skin thickening), and severe (tense subcutaneous fluid or thickened skin with lower eyelid festoons or upper lid fold remains rounded on downgaze). Conjunctival redness is classified as mild/equivocal, moderate (definite redness of < 50% bulbar conjunctiva excluding plica and caruncle), and severe (definite redness of ≥ 50%).
2. Eyelid measurements are mid pupil palpebral aperture, upper and lower lid retraction (distance from corneal limbus to eyelid margin), and levator function.
3. Proptosis is measured with Hertel's exophthalmometer.
4. Ocular motility is assessed by prism cover test at distance, torsion, monocular ductions, and the field of binocular single vision.
5. Corneal integrity and the risk of corneal breakdown assessed by the evaluation of lagophthalmos (asking patient to close their eyes as if asleep and using pen torch to see whether sclera or cornea is still visible) and Bell's phenomenon.
6. Optic neuropathy is judged on the basis of disc swelling or atrophy thought to be due to GO, decreased visual acuity, afferent pupil defect, and color vision, plus ancillary tests if necessary. Until further data is available, optic neuropathy may be assumed to be present if there is disc swelling or if two of the other clinical features are present. Impaired color perception carries more weight than other features except disc swelling [27].

#### 4.2.3. EUGOGO Classification of the Severity of the Ophthalmopathy

The management of patients with GO depends on the degree of severity of the ophthalmopathy, which is established according to the impact of the disease on the patient's quality of life and the risk of vision loss. The disease is classified as mild, moderate, severe, or sight-threatening as follows [3].

1. * Mild*: characteristics of GO have a minimum impact on the patient's life. They usually present one or more of the following signs:
  - Minor lid retraction (<2 mm).
  - Mild soft tissue involvement.
  - Exophthalmos < 3 mm (above the normal range for the race and gender).
  - Transient or no diplopia.
  - Corneal exposure responsive to lubricants.
2. *Moderate to severe*: patients without sight-threatening GO whose eye disease has sufficient impact on daily life to justify the risks of immunosuppression (if active) or surgical intervention (if inactive). Patients usually present one or more of the following signs:
  (i) Lid retraction (>2 mm).
  (ii) Moderate or severe soft tissue involvement.
  (iii) Exophthalmos ≥ 3 mm (above the normal range for the race and gender).
  (iv) Inconstant, or constant diplopia.
3. *Sight-threatening GO*: patients with dysthyroid optic neuropathy or corneal breakdown due to severe exposure. Other infrequent cases are ocular globe subluxation, severe forms of frozen eye, choroidal folds, and postural visual darkenings. This category warrants immediate intervention.

As a rule of thumb, it is considered that all patients who do not have a mild or a sight-threatening ophthalmopathy present a moderate-to-severe disease.

### 4.3. VISA or EUGOGO Classification: Which One to Use in Routine Clinical Practice?

Both VISA and EUGOGO systems provide not only a diagnostic classification, but also an assessment with practical implications for guiding the management of patients, something valuable compared to the NO SPECS classification.

The VISA classification follows a logical order from both an exploratory and management point of view (from vision to appearance). Symptoms and signs are clearly assessed and collected for each of the involved systems. Apart from a global severity grade every involved system is graded independently which adds an interesting value to assess the outcome of targeted treatments. Aside from this, the revised VISA classification evaluates disease activity based on the disease progression in any of the four parameters, either subjectively (patient documentation) or objectively (by clinical measurements). Therefore, an elevated inflammatory score provides evidence that the disease may be active, but it is not the only parameter evaluated to study activity [2]. In a similar way, the CAS assesses activity not only by the inflammation items but also with the progression in the last 3 items (parallel to V-S-A in the VISA classification). A disadvantage of including the “impaired function items” in the same score as the “soft tissue inflammation items” is that equal weight is given, for example, to redness of the eyelids and visual function worsening. As explained by Mourits et al. in the first publication of the modified CAS [24], they tried to adjust the CAS in such a way that some items had a double or triple weight, but this did not result in a more sensitive CAS. Therefore, they believed that the CAS should be used in combination with other parameters of disease activity, such as laboratory determinations. It is also important to have in mind that severe complications as DON can appear with low CAS scores.

Otherwise, while CAS is binary (absent/present: 0/1) for every item, the Inflammatory Score of VISA assigns a higher score for more severe forms of eyelid and conjunctival edema (0–2). Sometimes, it could be difficult to decide whether to score a patient to CAS 3 or 4 (and then candidate or not of intravenous immunosuppression) only for some subtle conjunctival or palpebral change. On the other hand, conjunctival redness is better defined in the CAS amended by EUGOGO than that in VISA, being scored only if it is moderate or severe, but not equivocal or mild. The Inflammatory Score of VISA also includes the diurnal variation item, a relevant clinical symptom referred by many patients in the active phase of the disease, while the CAS does not take it into account. The assessment of uniocular ocular excursion also has a different range in CAS and VISA-S (strabismus). While CAS considers a decrease in ocular excursion of > 8° in any direction for the definition of motility progression, VISA-S considers a decrease of >12°. Such figures come from the coefficients of repeatability found in different studies using several methods of assessing ocular ductions in patients with GO. The CAS definition of motility progression is based on the perimetry method, whereas VISA is from the light reflex technique. As demonstrated by Dolman et al. the coefficient of repeatability is of 12.2° for ductions measured either by perimetry or by the light reflex technique [26]. As the perimetry method is time consuming and requires a trained technician and an instrument that is increasingly unavailable, it seems that decreased ocular motility of >12° assessed by the light reflex technique is a better definition of motility progression. There are also differences between VISA and EUGOGO in grading the severity of ocular motility problems. While in the global score of VISA ocular motility involvement is evaluated with 2 items out of 5 (diplopia and restriction), only the diplopia but not the restriction is considered in the classification of severity according to EUGOGO.

EUGOGO classification of severity differentiates management categories which are very practical and of great help in deciding a specific management plan for the patient. However, there is no clear recording change in severity in their forms. EUGOGO does not differentiate between moderate and severe patients because they are all included in the same management plan. Likewise, VISA classification does not specify what is considered a mild, moderate, or severe appearance. On the other hand, VISA takes more into account the patient perception of her or his own illness in terms of assessing the grading and progression than does EUGOGO which is mainly a sign based classification. Interestingly, whereas several GO specific quality of life (QOL) questionnaires have been developed (GO quality of life questionnaire (GO-QOL); GO quality of life scale (GO-QLS); and TED quality of life questionnaire (TED-QOL)) all of them have shown only moderate correlation with disease severity, emphasising the discrepancy between objective and subjective assessments and the importance of measuring both in order to offer the best management plan to the patient [28].

As said previously, the EUGOGO and VISA classifications are not interchangeable. This is even more true when using these systems to assess the response rate to different treatments because, then, even the EUGOGO outcome criteria and the CAS give incongruent and incomparable response rates [29].

As both classifications have been devised to be used in clinical trials apart from clinical practice, they have some complexity in the extensive data to be collected. In any case, both ITEDS and EUGOGO groups provide downloadable forms for the first and follow-up visits. The ITEDS group might have developed a more elegant and user friendly form to complete in only one page. On the other hand, the assessment of CAS is easy and quick to collect in routine clinical practice.

As explained in the EUGOGO website, in July 2013 there was a meeting of members from the executive committees of both EUGOGO and ITEDS in Vancouver. The panel of experts agreed to proceed jointly to work on improved assessment systems. Until that work is developed, we propose to merge the Inflammatory Index of VISA and the CAS classification systems in a simplified “GO assessment activity checklist” in order to use it in routine clinical practice (Table 5). It joins the advantages of the more complete assessment of the Inflammatory VISA items, the separation of the progression items from the inflammatory items, and the simplicity of the CAS assessment.

## 5. Management Protocols of Graves' Ophthalmopathy

Treatment plan should be individually designed for each patient. An appropriate approach should be performed by a multidisciplinary team of ophthalmologists, endocrinologists, radiologists, and orbital surgeons [20]. It is vital to identify those patients who are likely to progress to serious complications such as restrictive strabismus or dysthyroid optic neuropathy before they develop.

Any patient with symptoms or signs of orbitopathy in the high-risk group (elderly, male, diabetic, or smoker), positive family history of orbitopathy, a recent history of progression, or any moderate inflammatory changes should be referred to the ophthalmologist within a few weeks. Cases with reported color or central visual loss, progressive diplopia, rapid deterioration in symptoms, or significant inflammatory scores should be urgently evaluated within a few days. Any patient who is undergoing radioiodine therapy for hyperthyroidism suspected of having active disease should be previously referred for an ocular examination to decide on the opportunity of prophylactic corticosteroid therapy [2].

Management and treatment modalities are decided according to the severity and activity of the disease. As mentioned, VISA management flow relies on the descending priority of treatment of the 4 affected functions in GO (impaired vision, soft tissue inflammation, ocular motility involvement, and appearance changes). On the other hand, the management categories of the EUGOGO severity classification are indeed practical and sharp. Here, we describe the management plan based on the Consensus Statement of the EUGOGO on management of GO of 2008 [3]. It has now been seven years from that consensus and numerous scientific evidences on GO treatment have been published since then. We summarize some of the current evidences on GO treatment and provide an update in the management of the disease (Table 6).

### 5.1. Measures for All Patients with Graves' Ophthalmopathy

#### 5.1.1. Restore Euthyroidism

Management of GO patients includes restoring and stabilizing thyroid function. Patients with uncontrolled thyroid dysfunctions are more likely to experience severe GO [30]. Furthermore, constant monitoring (every 4–6 weeks) of thyroid function is particularly important during the early stages of treatment.

Although controversial, the evolution of GO is likely not impacted by surgery or antithyroid [31–33]. Evidences exist that radioiodine worsens the active ocular disease in 15% of cases within the 6 months after the treatment. This risk may be reduced in patients with active GO by a short cycle (3 months) of orally delivered corticoids after the treatment (beginning with 0.3–0.5 mg of prednisone per kilogram daily and tapering the dose until withdrawal) and by avoiding postradioiodine hypothyroidism, which is also important in patients with inactive GO [3]. Interestingly, Lai et al. suggested that a lower dose of steroids may be equally effective in these cases [34].

It has not been until very recently that Stein et al. [35] have described that the risk of developing GO is substantially reduced in patients who undergo thyroidectomy compared with RAI ablation. They designed a retrospective longitudinal cohort study to specifically analyze the influence of the management of GD hyperthyroidism (treatment with antithyroid medications, exposure to RAI, or thyroidectomy) in the risk of developing GO. The hazard of developing GO was determined by multivariable Cox regression analysis among 8404 patients with newly diagnosed GD. During the follow-up, 740 (8.8%) enrollees developed GO. After adjustment for potential confounders, surgical thyroidectomy, alone or in combination with medical therapy, was associated with a 74% decreased risk for GO development (adjusted hazard ratio (HR), 0.26 (95% CI, 0.12–0.51)) compared with radioactive iodine therapy alone. Those patients not requiring treatment for hyperthyroidism exhibited a 73% decreased hazard of developing GO (adjusted HR, 0.27 (95% CI, 0.18–0.39)) relative to those treated with RAI alone. However, it is important to note that only 2 patients treated with RAI have been prescribed with corticosteroids after the treatment. Prospective studies are necessary to confirm these findings. In addition, the same group also found that after adjustment for covariates, enrollees with GD and statin use for more than 60 days in the previous year were associated with a 40% decreased hazard compared with those with less exposure to statins (adjusted HR, 0.60 (95% CI, 0.37–0.93)). This effect appears to be related to the anti-inflammatory actions of statins that are independent of their cholesterol-lowering properties.

#### 5.1.2. Conservative Measures

Patients should be advised to adopt general measures such as the use of artificial tears, sunglasses, and sleep with the head of the bed slightly elevated. Nocturnal ointment is of great benefit for incomplete eyelid closure provided that the cornea is protected.

#### 5.1.3. Smoking Cessation

Smoking is the most important risk factor amenable to modification in patients with GO and the risk is proportional to daily cigarette intake. Smokers with GO are more likely to develop a severe condition and have worse response to the immunosuppressant therapies [1, 30]. Xing et al. [36] have recently demonstrated that smoking, even past smoking, was an independent risk factor associated with impaired response to intravenous corticosteroids in patients with GO. Never smokers with active moderate-to-severe GO, who were treated with cumulative doses of 4.5 g intravenous methylprednisolone within 3 months, responded better than both active smokers and past smokers. Smoking patients did have more severe and active disease than never smokers. To exclude the interference of other factors, Xiang et al. performed a multivariable analysis and found a significant Odds Ratio (OR) of 12.4 (*P* = 0.035) for smoking patients to fail the treatment. This finding indicates that smoking compromised therapeutic effects not only through disease severity and activity but also through other independent mechanisms.

### 5.2. Measures for Patients with Mild Ophthalmopathy

Local measures are the mainstay therapy for patients with mild ophthalmopathy that generally have a self-limiting process. In the majority of studies which have investigated the natural history of GO in untreated patients, the orbitopathy improved in about a half of the patients, remained stable in about 35%, and worsened in approximately 15% [37]. Since up to 15% of the patients with mild disease may experience progression, a safe and well-tolerated preventive protocol as an alternative to the “wait and see” strategy seems to be justified. A recent study showed that a 6-month course with oral selenium (100 *μ*g twice daily) significantly improved quality of life, reduced ocular involvement, and slowed progression of the disease in patients with mild GO [38]. The use of oral corticosteroids is usually not recommended in patients with mild GO. Botulinum toxin injection may be considered to reduce upper lid retraction and is a valuable therapeutic option in active disease where definitive surgery remains contraindicated. Rehabilitative surgery (Müllerectomy or blepharoplasty) should be considered providing that the GO remains stable and inactive [39].

### 5.3. Measures for Patients with Moderate to Severe Ophthalmopathy

In these patients, eye disease has sufficient impact on daily life to justify the risks of immunosuppressant treatment (if active) or surgical intervention (if inactive).

#### 5.3.1. Immunosuppressive Medical Treatment and Orbital Radiotherapy

Only patients with active disease will respond to immunosuppressive treatments such as systemic corticosteroids or orbital radiation. These treatments have no benefit for patients in the quiescent phase in whom disease manifestations are the consequence of fibrotic changes in the orbital tissues [14].

A comparison of intravenous (IV) versus oral glucocorticoid administration has been reviewed by Zang et al. [40]. The overall response rate was 82% and 53.4% for intravenous and oral steroids, respectively. Pulses of IV steroids were associated with fewer side effects, shorter treatment course, and lower relapse risk compared with oral administration. The use of oral prednisone between IV pulses and its use in the tapering after IV glucocorticoids did not increase the response rate [40]. Oral corticosteroids might be considered when IV infusions are not logistically possible or if the patient prefers the oral route [41]. Oral corticosteroids might be also prescribed in some moderate to severe cases when the determination of activity is uncertain. A trial of therapy using a three-day course of oral prednisolone (50 mg) can determine whether clinical features show improvement, and, therefore, IV corticosteroids or radiotherapy may be indicated [2].

Although the optimum treatment protocol for patients with moderate to severe disease has yet to be defined in randomized controlled trials, a commonly used regimen is 500 mg methylprednisolone weekly for 6 weeks followed by 250 mg weekly for another 6 weeks, for a cumulative dose of 4.5 g [41]. This weekly therapy protocol of 4.5 g IV methylprednisolone cumulative dose is not only safer but is also more effective than a daily protocol (500 mg daily for 3 consecutive days per week for 2 weeks, followed by 250 mg daily for 3 consecutive days per week for another 2 weeks, and by tapering oral prednisone) [42]. If there is no clinical response, treatment with corticosteroids may be discontinued after the first 6 weeks [30, 40]. Prolongation of treatment after 12 weeks in patients who are responsive to corticosteroids should be related to disease severity and its impact on the quality of life, providing that the cumulative dose does not exceed 8 g and consecutive day-dosing is avoided. Fatalities with intravenous methylprednisolone in GO have only been reported when higher than 8 g cumulative dose was used. Severe adverse effects have occurred only in patients receiving daily and/or alternate single doses higher than 500 mg [40].

Assessment of liver morphology by sonography, liver function tests, detection of hepatitis viral markers, and autoantibodies have to be performed prior to the administration of IV treatment. Patients with recent hepatitis, liver dysfunction, severe cardiovascular morbidity, or severe hypertension must be excluded [40]. Liver enzymes, glucose levels, and blood pressure should be monitored monthly during treatment. Assessment of adrenal function may be advisable at the completion of the treatment with IV glucocorticoids.

In patients who are not responsive to corticosteroids, other treatments may be attempted. Though the efficacy of orbital radiotherapy alone for treatment of GO remains controversial, its combination with glucocorticoids is effective in early and active thyroid eye disease and has an acceptable safety profile [43]. Orbital radiotherapy (10–20 Gy in 10 sessions, over 2 weeks) is particularly effective in ocular motility involvement and, in some cases, in dysthyroid optic neuropathy. The group of Rootman and Dolman in Vancouver [44] has recently conducted a retrospective study to compare the risk of developing compressive optic neuropathy in 351 patients with active thyroid eye disease treated only with corticosteroids (144 patients) or with corticosteroids and orbital radiotherapy (105 patients). The main indications for offering orbital radiotherapy to patients already being treated with corticosteroids were development of significant restriction in ocular motility (88%); total cumulative dose of corticosteroids reaching unsafe levels over 8 g (17%); intolerance to corticosteroids (8.6%); and inadequate control of disease activity with corticosteroids (6.5%). At an average of 3.2 years follow-up, 17% of corticosteroids-only-treated group progressed to develop compressive optic neuropathy while 0% of corticosteroids-plus-radiotherapy-treated group (*P* < 0.0001) did. There were no known adverse effects secondary to orbital radiotherapy in this series. Although both groups experienced a significant reduction in periocular inflammation, the radiotherapy-treated group demonstrated a significantly greater improvement in diplopia and restriction in motility, supporting the idea that orbital radiotherapy combined with corticosteroids has an effective and sustained response and is protective against disease progression to restrictive myopathy and compressive optic neuropathy. Corticosteroids are effective at suppressing acute inflammation, but the response to corticosteroids is brief and may be poor or incomplete. Orbital radiotherapy may not show benefit for several days to weeks, but its effects last longer. Orbital radiotherapy is supposed to work through its nonspecific anti-inflammatory effects and the high radiosensitivity of lymphocytes infiltrating the orbital space and, hence, by reducing the secretion of proinflammatory cytokines from activated lymphocytes. Moreover, orbital radiotherapy may target orbital fibroblasts inducing terminal differentiation in progenitor fibroblasts, suppressing the downstream consequences of fibroblast activation by reducing their capability to synthesize and secrete glycosaminoglycans [44].

Thus, radiotherapy should be considered a second-line treatment, when the first course of steroids has produced only a partial response and the disease is still active. It should be avoided in patients younger than 35 years of age or in patients with diabetes or severe hypertension [45].

Some patients seem to have a phase of activity which lasts longer than usual. They may have a recurrence of inflammatory signs of orbitopathy after withdrawal of corticosteroid treatment because of onset of steroid side effects or intolerance. These patients can be candidates for a trial of treatment with methotrexate (weekly dose of 7.5 mg to 10 mg orally administered and fractionated) [46].

Alternatively, patients who are nonresponsive to corticosteroids may be treated with combination therapy of cyclosporin A (5 mg/kg/day in 2 doses plus oral glucocorticoids), azathioprine, or specific monoclonal antibody agents. In preliminary clinical trials, rituximab significantly reduced the inflammatory activity and severity of the ophthalmopathy in patients with active eye disease. However, two recent randomized trials on the efficacy of rituximab in moderate to severe GO reported conflicting results [47, 48]. The reasons for this disagreement are unclear but may be related to the differences in the study designs. The rationale is also strong for the study of other immunomodulatory agents in GO, including those targeting receptors for IL-1, IL-6, and TNF, modulating costimulatory pathways or decreasing leukocyte recruitment into the orbit [49]. There are some evidences in short series reports of the efficacy of other molecules such as tocilizumab, adalimumab, or etanercept [50, 51]. Other promising treatments such as the production of TSH-R antagonists, either as monoclonal TSH-R-blocking antibodies or as small-molecule-ligand antagonists of TSH-R, are being developed [52].

#### 5.3.2. Surgical Treatment

Once the disease has become inactive, several rehabilitative surgical procedures for patients with moderate to severe ophthalmopathy may be used. Before offering surgery, patients should show evidence of disease quiescence over a period of at least 6 months.

Available procedures include orbital decompression for disfiguring proptosis [53]; strabismus surgery for symptomatic ocular motility restriction; eyelid recession for eyelid retraction causing lagophthalmos, exposure keratitis, and disfigurement; and blepharoplasty for excessive soft tissue prominence of the eyelids [14, 39, 54]. If necessary, orbital decompression must be first addressed because of its influence on ocular motility and lid width followed by strabismus surgery and, finally, eyelid surgery.

Many different techniques and approaches have been described for orbital decompression surgery, including 1-, 2-, and 3-wall decompression with orbital fat removal depending on the degree of exophthalmos. Different combinations of medial, inferior, and lateral wall decompression have been used as areas of bone removal. Proptosis regression after surgery varied from 5.6 to 6.5 mm after 3-wall decompression and from 3.2 to 4.8 mm after 2-wall decompression [54, 55]. Surgically induced diplopia is the most common complication of orbital decompression, with the highest rates of 38%–60% reported with inferomedial decompression, possibly as a result of inferomedial shift of the globe after removing the inferomedial strut. Balanced orbital decompression may reduce the incidence of postoperative diplopia by producing a more equivalent displacement of the medial and lateral soft tissues into the surrounding space. New techniques such as deep lateral wall orbital decompression [53] or modified endoscopic medial orbital fat decompression [56] carry a lower risk of morbidity associated to ocular motility problems while providing a significant proptosis reduction.

The goal of extraocular muscle surgery in patients with GO is to restore binocular single vision in the primary position at distance and near (reading position). Residual double vision may persist in peripheral positions of gaze. The basic concept of most operations is to recess the fibrotic muscles in order to correct ocular ductions. Muscle resections should be avoided since any restriction is likely to be aggravated if a muscle is shortened. Most vertical deviations can be corrected by single inferior rectus recessions due to high-dose effect. Dose effects for medial rectus recessions are lower and bilateral medial rectus muscle is often required to treat horizontal strabismus [54].

Lid retraction of both upper and lower eyelids is probably the most common feature of GO. Surgery is recommended for significant upper lid retraction of >1 mm, asymmetry of palpebral apertures, or lateral (temporal) flare. Surgery for upper lid retraction is divided into the anterior approach through an eyelid crease incision where the levator aponeurosis and Müller's muscle are disinserted from the tarsus until appropriate height of the eyelid is achieved and a posterior approach through the conjunctiva and Müller's muscle. In most cases, the use of implants is not necessary [54].

Lower lid lengthening is indicated in lower lid retraction. It mainly occurs in cases in which the ligamentum capsulopalpebrale to the lower lid retractors has not been disinserted when an inferior rectus recession has been performed. In lower lid retraction repair, the conjunctiva and lower lid retractors are detached from the edge of the tarsus through a posterior approach and a spacer (auricular cartilage, hard palate mucosa, expanded polyethylene microplates, autogenous tarsus transplants, porcine acellular dermal matrix, donor sclera, or pericardium) is placed between the retractors and tarsus [54].

Upper and/or lower eyelid blepharoplasty is frequently needed as the last step in the functional and cosmetic rehabilitation of GO patients.

### 5.4. Measures for Patients with Sight-Threatening Ophthalmopathy

Patients with sight-threatening ophthalmopathy due to dysthyroid optic neuropathy must be treated urgently. High-dose intravenous glucocorticoids are the recommended first-line treatment for DON (3 × 500 mg–1 g on consecutive days within one week; if necessary, repeated the following week). If the response is insufficient after 1-2 weeks, or the dose/duration of steroid treatment required induces significant side effects, orbital decompression (deep medial orbital wall decompression including posterior ethmoidal cells near the orbital apex) should be carried out promptly [54, 57]. Immediate surgical decompression as first-line therapy has not resulted in a better outcome than the use of intravenous steroids followed by decompression in those patients with no response [58].

**Figure 4:**
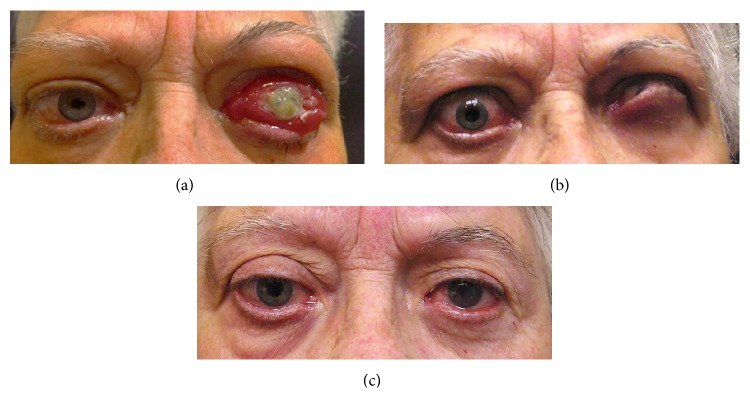
Threatening-to-vision GO. (a) Initial presentation of a patient with threatening-to-vision GO. LE: corneal breakdown, chemosis, conjunctival redness, eyelid swelling, swollen caruncle, retrobulbar ache at rest and with gaze, diurnal variation (inflammatory score: 9/10), proptosis > 2 mm, optic neuropathy, and extraocular muscle restriction (3/3 progression score). (b) Appearance after methylprednosolone IV treatment, amniotic membrane transplant, and lateral tarsorrhaphy in LE. (c) Appearance after bilateral orbital decompression and levator recession surgery (Dr. Barrio-Barrio and Dr. Fernandez-Hermida performed the surgical procedures).

Depending on the severity of the exophthalmos, cases of corneal exposure keratopathy could be treated with aggressive topical lubrication, moisture chamber, botulinum toxin, levator recession surgery, tarsorrhaphy, or even orbital decompression in very severe cases of exophthalmos which impede lid closing. Intravenous methylprednisolone should be administered prior surgery if the disease is active (Figure 4).

## 6. Conclusions

GO is a complex inflammatory disorder that is better managed by a multidisciplinary team. Early detection of sight-threatening ophthalmopathy and identification of risk factors for severe outcomes are critical. An appropriate assessment of both severity and activity of the disease warrants an adequate treatment. The VISA and EUGOGO grading systems have been demonstrated as invaluable tools in the assessment and management of GO patients. A simplified “GO activity assessment checklist” would help the physicians involved in the management of these patients in routine clinical practice. Intravenous glucocorticoids remain the treatment of choice for active moderate to severe disease. Orbital radiotherapy in combination with glucocorticoids is considered a second-line treatment which is particularly effective when ocular motility is involved. Immediate surgical decompression may be needed in cases of dysthyroid optic neuropathy. Several rehabilitative surgical procedures may be necessary once the disease has become inactive. New treatment modalities such as specific monoclonal antibodies, TSH-R antagonists, and other immunomodulatory agents show a promising outcome for GO patients.

## Figures and Tables

**Table 1:** NO SPECS modified classification [22].

Class	Grade	Suggestions for grading
0		No physical signs or symptoms

I		Only signs

II		Soft tissue involvement
	0	Absent
	a	Minimal
	b	Moderate
	c	Marked

III		Proptosis (3 mm or more of normal upper limits with or without symptoms)
	0	Absent
	a	3 or 4 mm over upper normal
	b	5 to 7 mm increase
	c	8 mm increase

IV		Extraocular muscle involvement (usually with diplopia)
	0	Absent
	a	Limitation of motion at extremes of gaze
	b	Evident restriction of motion
	c	Fixation of a globe or globes

V		Corneal involvement (primarily due to lagophthalmos)
	0	Absent
	a	Stippling of cornea
	b	Ulceration
	c	Clouding, necrosis, and perforation

VI		Sight loss (due to optic nerve involvement)
	0	Absent
	a	Disc pallor or choking, or visual field defect, vision 20/20–20/60
	b	The same, but vision 20/70–20/200
	c	Blidness, vision less than 20/200

**Table 2:** Clinical Activity Score (CAS) (amended by EUGOGO after Mourits et al.). One point is given for the presence of each of the parameters assessed. The sum of all points defines clinical activity: active ophthalmopathy if the score is above 3/7 at the first examination or above 4/10 in successive examinations.

	For initial CAS, only score items 1–7
1	Spontaneous orbital pain
2	Gaze evoked orbital pain
3	Eyelid swelling that is considered to be due to active GO
4	Eyelid erythema
5	Conjunctival redness that is considered to be due to active GO
6	Chemosis
7	Inflammation of caruncle OR plica

	Patients assessed after follow-up (1–3 months) can be scored out of 10 by including items 8–10

8	Increase of >2 mm in proptosis
9	Decrease in uniocular ocular excursion in any one direction of >8°
10	Decrease of acuity equivalent to 1 Snellen line

**Table 3:** VISA Inflammatory Index (I) (Dolman and Rootman 2006 [25], ITEDS modified). Patients with moderate inflammatory index (less than 4 of 10) are managed conservatively. Patients with high scores (above 5 of 10) or with evidence of progression in the inflammation are offered a more aggressive therapy.

Sign or symptom	Score
Caruncular edema	0: absent 1: present

Chemosis	0: absent 1: conjunctiva lies behind the grey line of the lid 2: conjunctiva extends anterior to the grey line of the lid

Conjunctival redness	0: absent 1: present

Lid redness	0: absent 1: present

Lid edema	0: absent 1: present but without redundant tissues 2: present and causing bulging in the palpebral skin, including lower lid festoon

Retrobulbar ache At rest With Gaze	0: absent; 1: present 0: absent; 1: present

Diurnal variation	0: absent; 1: present

**Table 4:** Protocol to assess the severity of Graves' ophthalmopathy (EUGOGO). Some of the signs may be assessed by comparison with the image atlas provided by the EUGOGO (http://www.eugogo.eu/).

Soft tissues	*Eyelid swelling* (i) Absent (ii) Mild: none of the features defining moderate or severe swelling are present (iii) Moderate: definite swelling but no lower eyelid festoons and in the upper eyelid the skin fold becomes angled on a 45° downgaze (iv) Severe: lower eyelid festoons OR upper lid fold remains rounded on a 45° downgaze
	*Eyelid erythema* (i) Absent (ii) Present
	*Conjunctival redness* (i) Absent (ii) Mild: equivocal or minimal redness (iii) Moderate: <50% of definite conjunctival redness (iv) Severe: >50% of definite conjunctival redness
	*Conjunctival edema* (i) Absent (ii) Present: separation of conjunctiva from sclera present in >1/3 of the total height of the palpebral aperture or conjunctiva prolapsing anterior to grey line of eyelid
	*Inflammation of caruncle or plica semilunaris* (i) Absent (ii) Present: plica is prolapsed through closed eyelids or caruncle and/or plica are inflamed

Eyelid measurements	*Palpebral aperture (mm)* *Upper/lower lid retraction (mm)* *Levator function (mm)* *Lagophthalmos* (i) Absent (ii) Present *Bell's phenomenon* (i) Absent (ii) Present

Proptosis	Measurement with Hertel's exophthalmometer. Recording of intercanthal distance.

Ocular motility	*Prism cover test* *Monocular ductions* *Head posture* *Torsion* *Field of binocular single vision*

Cornea	*Corneal integrity* (i) Normal (ii) Punctate keratopathy (iii) Ulcer (iv) Perforation

Optic neuropathy	(i) Visual acuity (Logmar or Snellen) (ii) Afferent pupil defect (present/absent) (iii) Colour vision (iv) Optic disc assessment: normal/atrophy/edema

**(a):** 

Inflammatory signs and symptoms^*∗*^			
	0	1	2
Diurnal variation (0)-(1)	Absent	(i) Inflammation worse with the head dependent after sleep or (ii) worsening of diplopia at morning	

Retrobulbar ache at rest (0)-(1)	Absent	Present	

Retrobulbar ache with gaze (0)-(1)	Absent	Present	

Lid edema (score worst eyelid) (0)-(1)-(2)	(i) Absent or (ii) mild or (iii) not thought to be due to active GO	Present but without redundant tissues	Present and causing bulging in the palpebral skin (tense subcutaneous fluid): (i) upper lid fold remains rounded on downgaze or (ii) lower lid festoon

Lid redness (score worst eyelid) (0)-(1)	(i) Absent or (ii) not thought to be due to active GO	Present	

Conjunctival redness (0)-(1)	(i) Absent or (ii) equivocal or (iii) mild or (iv) not thought to be due to active GO	Diffuse redness, covering at least one quadrant assessed without slit-lamp at 1 meter from the patient	

Chemosis (0)-(1)-(2)	(i) Absent or (ii) conjunctivochalasis	Separation of conjunctiva from sclera present in >1/3 of the total height of the palpebral aperture Conjunctiva behind posterior to the grey line	Conjunctiva anterior to the grey line

Inflammation of caruncle OR plica (0)-(1)	Absent	(i) Plica is prolapsed through closed eyelids or (ii) caruncle and/or plica are inflamed	

Total inflammatory score: /10			

^**∗**^Score worst eye.

**(b):** 

Progression symptoms (changes in the previous 1–3 months)		
	0	1
Optic Neuropathy (0)-(1)	Same or Better	Disc swelling or atrophy thought to be due to GO, or 2 of the following: (i) Decreased visual acuity equivalent to 1 Snellen line (ii) Afferent pupil defect (iii) Impaired colour perception

Extraocular muscle ductions (0)-(1)	Same or Better	Decrease in uniocular ocular excursion in any one direction of ≥12°

Proptosis (0)-(1)	Same or Better	Increase of ≥2 mm in proptosis

**Table 6:** Orientative therapeutic protocol for Graves' ophthalmopathy (see text for bibliographic references).

All patients	Restore euthyroidism	
	Avoid smoking	
	Conservative local measures	
Severity	Activity	
	Active	Nonactive
Mild	(i) Artificial tears (ii) Sunglasses (iii) Head of the bed slightly elevated (iv) Selenium (200 *μ*/g daily × 6 months) (v) Fresnel-type prisms (vi) Botulinum toxin in Müller muscle	(i) Artificial tears (ii) Prisms (iii) Botulinum toxin in Müller muscle (iv) Surgical Müllerectomy (v) Blepharoplasty

Moderate-severe	(i) Intravenous methylprednisolone: 1st, 500 mg/week × 6 weeks 2nd, 250 mg/week × 6 weeks 3rd, if activity persists: consider prolongation of treatment up to 8 g of maximum cumulative dosage 4th If non responsive after 6 weeks, change the treatment (ii) Patients resistant to glucocorticoids: (a) Association of cyclosporin A (5 mg/kg/day in 2 doses) plus oral glucocorticoids, methotrexate (7,5–10 mg/week), tocilizumab (8 mg/kg/every 4 weeks), and Rituximab (500 mg–1000 mg), (b) If muscular involvement predominates: orbital radiotherapy (20 Gy) (not in <35 years or diabetic patients) (iii) Consider botulinum toxin in extraocular muscles if with diplopia (medial rectus or inferior rectus)	1st, orbital decompression (2 or 3 walls depending on the degree of exophthalmos) 2nd, surgery for strabismus (stability of 6-month deviation angle. Muscular recessions) 3rd, palpebral surgery (i) Palpebral retraction: levator recession surgery, retractors for the lower eyelid. (ii) Blepharoplasty of upper eyelids, lower eyelids, or both.

Threat to vision		
Dysthyroid optic neuropathy	Methylprednisolone 1 g intravenously × 3 days, repeat after a week If nonresponsive: urgent orbital decompression. (+/− glucocorticoids intravenously if still active +/− radiotherapy)	Urgent deep orbital medial wall decompression
Severe exposure keratopathy	Intravenous methylprednisolone when relevant orbital inflammation; palpebral closure, lubrication, tarsorrhaphy, botulinum toxin in Müller muscle, and orbital decompression if other measures are inefficient	Lateral tarsorrhaphy, orbital decompression, amniotic membrane transplant, and corneal transplant
